# Effectiveness of Antihyperglycemic Effect of* Momordica charantia*: Implication of T-Cell Cytokines

**DOI:** 10.1155/2017/3707046

**Published:** 2017-11-28

**Authors:** Rufine Fachinan, Akadiri Yessoufou, Magloire Pandoua Nekoua, Kabirou Moutairou

**Affiliations:** Laboratory of Cell Biology and Physiology, Department of Biochemistry and Cellular Biology, Faculty of Sciences and Technology (FAST) and Institute of Applied Biomedical Sciences (ISBA), University of Abomey-Calavi, 01 BP 918 Cotonou, Benin

## Abstract

**Background/Objective:**

We investigate the effect of antidiabetic* Momordica charantia* fruit juice on T cells' differentiation, through plasmatic cytokine quantification in type 1 diabetic rats (T1D).

**Methods:**

Male* Wistar* rats were rendered diabetic by the injection of five low doses of streptozotocin. Then, animals were treated with* Momordica charantia* fruit juice for 28 consecutive days. Plasmatic levels of Th1 interleukin- (IL-) 02 and interferon- (IFN-) *γ*, Th2 (IL-4), and regulatory (IL-10) cytokines were determined in rats.

**Results:**

We observed that fruit juice induced a significant decrease in blood glucose of T1D rats. Besides, the concentrations of IL-2 and IFN-*γ* significantly increased while those of IL-4 and IL-10 diminished in diabetic rats compared to control animals. Interestingly, after treatment with* Momordica charantia* fruit juice, IL-4 and IL-10 levels significantly increased in diabetic rats, while IL-2 and IFN-*γ* concentrations decreased, suggesting a Th2 phenotype in these animals. Phytochemical analysis of the fruit juice revealed the presence of tannins, flavonoids, and coumarins, compounds which possess antioxidant activity.

**Conclusion:**

This study shows that* Momordica charantia* fruit juice, by lowering the hyperglycemia, induced a shift of proinflammatory Th1 phenotype in T1D rats towards a favorable anti-inflammatory Th2 status. These effects might be due to the presence of antioxidant compounds in the juice and confirms the use of this plant in the treatment of autoimmune type 1 diabetes.

## 1. Background

Medicinal plants in developing countries are often used for primary health care as an alternative option to modern synthetic drugs that are more costly. Most of these plants are empirically used by the populations and are still suffering from the lack of scientific investigation. For our contribution, we have previously undertaken studies and demonstrated the antihyperglycemic effects of three African medicinal plants in diabetic pregnancy in rats [1]. Very recently, we have also shown the immune-modulatory effect of* Momordica (M.) charantia* [2], a plant commonly used in Africa and south Asia for diabetes treatment. During several decades,* M. charantia* is one of the plants which have gained most attention from several researchers [2–10].* M. charantia* also called “bitter melon” or African cucumber is a plant of Cucurbitaceae family widely cultivated in tropical and subtropical regions and is commonly used in Mediterranean traditional medicine for its antidiabetic properties and antihyperglycemic, antitumor, anti-inflammatory, and cytotoxic activities [3–9]. In addition,* M. charantia* fruit juice has been shown to induce regeneration of pancreatic beta cells in streptozotocin- (STZ-) induced diabetic rats [8]. Moreover,* M. charantia* (karela) fruit extract has exhibited hypotriglyceridemic and hypocholesterolemic effects of antidiabetes in STZ-induced diabetic rats [9]. It also significantly stimulated both the storage of glycogen in the liver [10] and insulin secretion by *β* cells isolated from the islets of Langerhans [11]. Nonetheless, the exact mechanism of action of this plant remains unclear. Besides, several studies have demonstrated the role of the immune system and inflammation in the pathogenesis of different forms of diabetes [12, 13]. This role of the immune system is devoted to the implication of T-lymphocytes [12], the principal mediators of immune responses in health and disease. Indeed, type 1 diabetes is characterized by the autoimmune destruction of pancreatic beta cells by autoreactive leukocytes through the actions of cytokines or cell-cell contact [12–15]. For instance, it is well known that naïve T-helper cell (Th0) can differentiate into several specific subsets (Th1, Th2, Th9, Th17, Th22, Treg cells, etc.) under the influence of cytokines [16]. Th1 cells, producing proinflammatory cytokines (IL-2, IL-12, and IFN-*γ*), support cell-mediated immunity, while Th2 cells, secreting anti-inflammatory cytokines (IL-4, IL-5, and IL-13), support humoral immunity and antagonize the inflammatory actions of Th1 cells [16–19]. IL-10 is produced by several cells (Treg cells, CD4+ Teff cells, and Breg cells) and more and more it is classified as a regulatory cytokine [20–24].

As mentioned above, we have found in our very recent study that the filtered* M charantia* fruit juice (saponins-depleted fruit juice) elicited immunosuppressive and Th2-inducing phenotype on human T-lymphocytes* in vitro *[2], suggesting that* M. charantia* fruit juice could have beneficial effect in autoimmune type 1 diabetes. Thus, we were prompted to investigate the effect of such juice* in vivo* in STZ-induced diabetic rats. Therefore, the aim of this study was to investigate the effect of* M. charantia* fruit juice on T-lymphocyte differentiation in STZ-induced type 1 diabetes.

## 2. Materials and Methods

### 2.1. Plant Materials' Description and Collection

Fresh fruits of* M. charantia* were collected from the southeastern part of Benin from mid-July to mid-August. The temperature at this time was at 28.2°C (ASECNA, Air Navigation and Security Agency, Dangbo station, Ouémé department). This period was at the end of the long rainy season (mid-March to mid-July). The soil is hydromorphic lateritic on clay sediments (reference: Carte pédologique de reconnaissance à 1/200000, Feuille de Porto-Novo 1975, Benin) and the plant adapts to this kind of soil. The plant was identified by the Principal Botanist of the National Herbarium of Benin of the University of Abomey-Calavi, where the voucher specimens were deposited under the following number:* Momordica charantia* L. Cucurbitaceae: AP2033/HNB.

### 2.2. Plant Juices' Preparation

*M. charantia* fruit juice was prepared as we have previously described [2]. Briefly, 100 g of fresh fruits were manually ground and pressed with 100 ml of sterilized distilled water to obtain fruit juice according to slightly modified methods of Raza et al. [8]. The debris was removed by passing the juice through a clean cotton column in a funnel. The obtained fruit juice was then filtered on filter paper (Prolabo filter paper for Ashless analysis, diameter 150 mm, Paris, France) and used for animal treatments. Some parts of juice were distributed in aliquots and frozen at −80°C for other uses.

### 2.3. Diabetes Induction

Male* Wistar* rats of age of 2 to 3 months (200–250 g) were obtained from animal facilities of Institute of Applied Biomedical Sciences (ISBA). They were housed in wood chip-bedded plastic cages and maintained in a controlled environment (12 : 12 hours light/dark cycle) and temperature (25°C). Animals were divided into experimental groups which consisted each of ten rats. For diabetes induction, rats were rendered diabetic by intraperitoneal administration of five low doses of streptozotocin (40 mg/kg body weight, in 0.1 M citrate buffer, pH 4.5). Control animals were injected with the citrate buffer.

### 2.4. Animal Treatment by* Momordica charantia* Fruit Juice

As we mentioned above, animals were injected with STZ for 5 days. Then,* Momordica charantia* fruit juice was orally administrated to diabetic or control animals (10 ml/kg body weight) for 28 consecutive days (four weeks) starting from the 7th day (one week) after the last day of STZ injection. This juice did not show any cell toxicity, as we have recently observed [2]. To determine glycemia during the experimentation, blood was collected after an overnight fasting by cutting off the tip of the tail and squeezing it gently. Then, fasting glycemia was measured using One Touch ULTRA Glucometer (Life Scan, Johnson and Johnson, USA). At the last day of treatment with fruit juice, rats were fasted overnight and anaesthetized with pentobarbital (60 mg/kg body weight), as described previously [20, 21]. The abdominal cavity was opened, and blood was drawn from the abdominal aorta in tube containing potassium oxalate and sodium fluoride or heparin. Plasma from oxalate tubes was obtained by low-speed centrifugation (1000*g*, 20 min) and immediately used for glucose determinations by glucose oxidase method using glucose analyzer (Beckman Instruments Fullerton, CA, USA). Plasma samples from heparin tube were distributed in aliquots and stored at −80°C for future measurements of Th1 and Th2 cytokine concentrations. Repeated freeze-thaw cycles were avoided. The general guidelines for the care and use of laboratory animals, recommended by the Council of European Economic Communities, were followed. The experimental protocol was approved by the Regional Ethical Committee (Comité d'Ethique de l'Expérimentation Animale of University of Bourgogne, Dijon, France; Researcher Authorization number 21 CAE 069).

### 2.5. Determination of Th1/Th2 Cytokine Concentrations in the Plasma

In order to test the effects of fruit juice on T-helper cell phenotype, we quantified the concentrations of T-cell differentiation cytokines (IL-2, IL-4, IL10, and IFN-*γ*) in the animal plasma, using Abcam Rat ELISA kit for IL-2, IL-4, and IL-10 (Abcam, Cambridge, USA) and Bio-Legend Rat LEGEND MAX™ ELISA kit for IFN-*γ* (Bio-Legend, San Diego, CA, USA). The manufacturer's instructions were followed for the different assays. The minimum detectable concentrations were 0.1 ng/mL, 1.5 pg/mL, 4.89 pg/mL, and 3.2 pg/mL for IL-2, IL-4, IL-10, and IFN-*γ*, respectively. At least 90% of rats had detectable levels of all cytokines except for IL-2 which is not detectable in roughly 15% of rats. The theoretical values of cytokines assigned to rats with circulating levels of cytokines below the limit of sensitivity of the assay were “zero.” We also calculated the Th1/Th2 cytokine ratios, determined as IL-2/IL-4 and IFN-*γ*/IL-4 (Table 1).

### 2.6. Phytochemical Analysis of Plant Juice

Chemical compounds of* M. charantia* fruit juice (Table 2) were investigated using the methods of Ciulei [25] based on colorimetric reactions and differential precipitations. Briefly, the juice was evaporated under vacuum at 50°C (Rotavapor) to obtain powders which were subjected to the determination of different compounds as described elsewhere [26].

### 2.7. Antioxidant Activities of* M. charantia* Fruit Juice

The antioxidant status of fruit juice was assessed by determining the ability of fruit juice to scavenge a free radicals' generator, the 2,2-diphenyl-1-picrylhydrazyl radical (DPPH). The antioxidant activity was determined according to the method previously described [2]. All tests were performed in triplicate. DPPH radical inhibition percentage was calculated according to the following formula: inhibition (%) = [(AB − AS)/AB] × 100, where “AS” is the sample (tested extract solution) absorbance and “AB” is the blank absorbance.

### 2.8. Statistical Analysis

Data are expressed as mean ± SEM. Mean values were compared by two-way ANOVA, followed by LSD test. Differences were considered significant when *p* < 0.05.

## 3. Results

### 3.1. Effects of* Momordica charantia* Fruit Juice on Glycemia in T1D Rats

**Figure 1:**
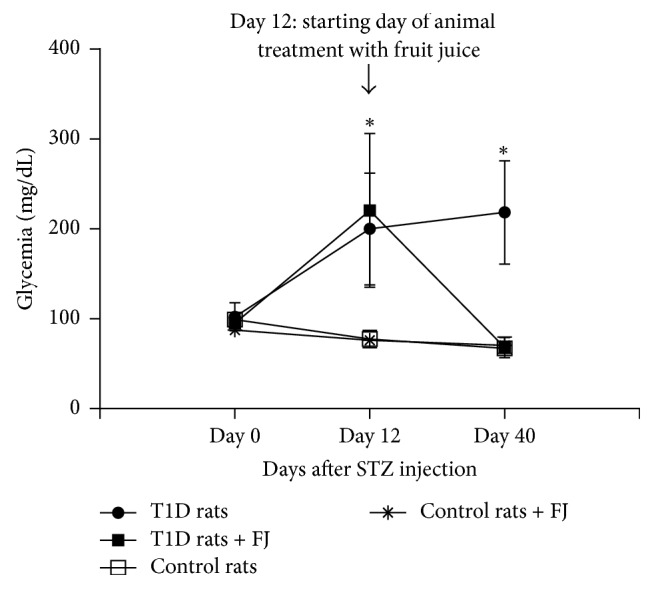
Glycemia was measured during the experiment in animals treated or not with* Momordica charantia* fruit juice (FJ). Rats were rendered diabetic by intraperitoneal injection of five low doses of streptozotocin (STZ). From the 7th day (1 week) after the last day of STZ (diabetic rats) or citrate buffer (control rats) injection, animals were treated with* Momordica charantia *fruit juice for 28 consecutive days (four weeks). Then, rats were fasted overnight and anaesthetized and blood was collected from the abdominal aorta for glycemia and other experiments. Values are means ± SD; *n* = 10 rats per group. T1D: type 1 diabetic rats. ^*∗*^*p* < 0.05 indicates significant difference between diabetics and control animals.

Glycemia of STZ-induced type 1 diabetic rats was significantly higher than that of control animals. However, the glycemia was significant decreased to normal level in diabetic rats treated with* M. charantia* fruit juice (Figure 1). There was no significant difference in the glycemia of control animals treated or not with* M. charantia* fruit juice (Figure 1).

### 3.2. *Momordica charantia* Fruit Juice Modulates* In Vivo* Th1/Th2 Cytokines

**Figure 2:**
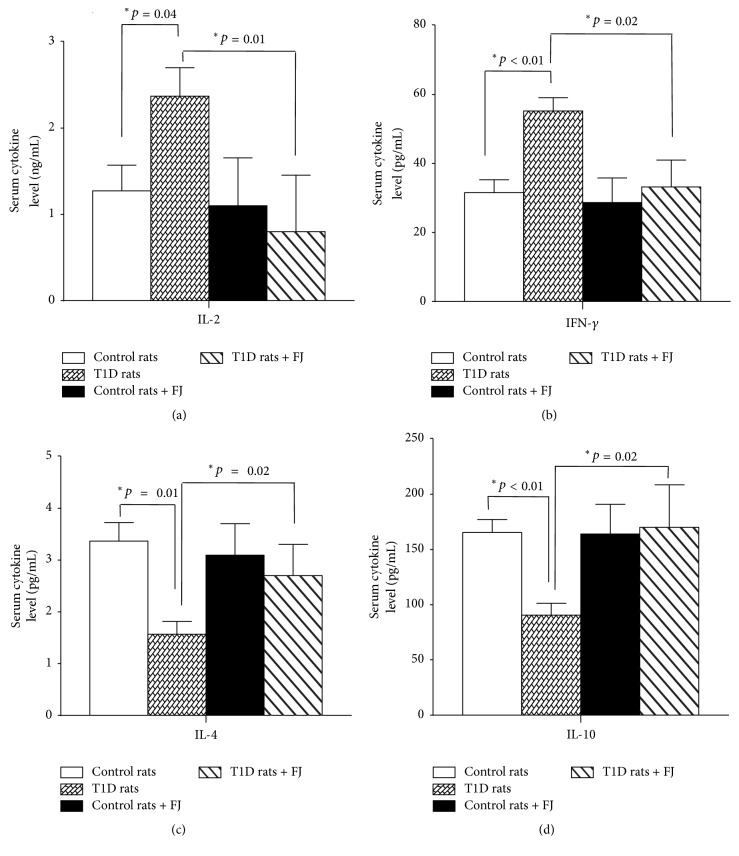
Th1 and Th2 cytokine concentrations in the plasma of T1D and control rats. Cytokine concentrations were determined in animals after the 28 consecutive days (four weeks) of treatment with* Momordica charantia *fruit juice, as described in* Materials and Methods*. (a) IL-2, (b) IFN-*γ*, (c) IL-4, and (d) IL-10 plasma concentrations. Values are means ± SEM; *n* = 10 rats per group of animals. Data were analyzed by two-way ANOVA followed by the Least Significant Difference test. (^*∗*^*p* < 0.05) indicates significant difference between groups.

Plasmatic levels of IL-2 and IFN-*γ* (Th1 cytokines) were significantly higher in T1D rats than in controls (*p* = 0.04 and *p* < 0.01, resp.) (Figures 2(a) and 2(b)). Concomitantly, the concentrations of IL-4 (Th2 cytokine) and IL-10 (regulatory cytokine) were significantly lower in the plasma of T1D rats than that of control rats (*p* = 0.01 and *p* < 0.01, resp.) (Figures 2(c) and 2(d)). However, treating the T1D rats with* M. charantia* fruit juice diminished the IL-2 and IFN-*γ* concentrations (*p* = 0.01 and *p* < 0.01, resp.) and enhanced IL-4 and IL-10 levels (*p* = 0.01 and *p* < 0.01, resp.), as compared to untreated T1D rats (Figures 2(a), 2(b), 2(c), and 2(d)). There was no significant difference in the levels of these cytokines between treated and untreated control animals with fruit juice (Figures 2(a), 2(b), 2(c), and 2(d)). The Th1/Th2 cytokine ratios, determined as IL-2/IL-4 and IFN-*γ*/IL-4, were significantly shifted from a Th2 phenotype in control rats to Th1 status in diabetic animals. In contrast, these ratios were shifted from Th1 in diabetic rats to Th2 in diabetic rats treated with* M. charantia *fruit juice (Table 1). No difference was observed in the ratios of control animals treated or not with fruit juice (Table 1).

### 3.3. *Momordica charantia* Fruit Juice Is Rich of Tannins, Flavonoids, and Coumarins

In attempt to link chemical composition of the juice with the observed effects on glycemia and* in vivo* cytokines' production, we performed phytochemical analysis of the plant juice. The results showed that* M. charantia* fruit juice was rich of polyphenols (tannins, flavonoids, and coumarins). Alkaloids and cyanogenic derivatives, saponins and anthraquinones, were undetectable in the juice (Table 2).

### 3.4. *Momordica charantia* Fruit Juice Exhibits Antioxidant Activities

**Figure 3:**
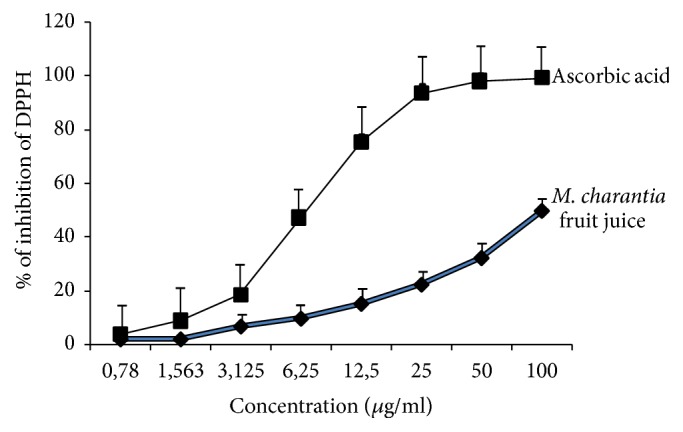
Antioxidant activities of* Momordica charantia* fruit juice. Values are means ± SEM. Antioxidant capacity of plant fruit juice was determined in a solution of fruit juice (0.78–100 *μ*g/ml) as described in the* Materials and Methods*. Each value represents the mean of three determinations.

Antioxidant capacities determined as DPPH radical scavenging activities of* M. charantia* fruit juice increased gradually in a dose-dependent manner (Figure 3), from 0.78 *μ*g/ml to 100 *μ*g/ml, with the highest antioxidant activity at 100 *μ*g/ml (1.93 ≤ IP% ≤ 49.95), of which substantial inhibition percentage (IP%) was roughly 50%, as compared to that of ascorbic acid.

## 4. Discussion

*M. charantia* is one of the antidiabetic plants used, without knowing their exact physiological mechanism of action. Therefore, the aim of this study was to investigate the effects of* M. charantia* fruit juice on hyperglycemia through its effects* in vivo *on T-cell differentiation in type 1 diabetic rats.

In the present study, we induced type 1 diabetes by injecting five low doses of STZ. First, we would like to state that diabetes induced with* multiple* low doses of STZ represents a good model of autoimmune type 1 diabetes. Indeed, STZ when administered at a high single dose induces diabetes by the direct toxic effects on pancreatic *β*-islet cells [12]. However, when STZ is administered at low doses during five consecutive days, it induces mild type 1 diabetes, through a T-lymphocyte-dependent process, an autoimmune destruction of pancreatic *β* cells mediated by both CD4+ and CD8+ T cells [13, 14]. The autoimmune process commences with the infiltration of T cells in pancreas tissue roughly on the 2nd day after the last injection of STZ [12–14]. Diabetes occurs on the 7th day and the glycemia becomes maximal after two weeks from the last injection of STZ [12–14]. According to the reports above, we started the treatment of animals with* M. charantia *fruit juice, orally administrated to diabetic or control animals, from the 7th day after the last injection of STZ.

In this study, we observed that* M. charantia* fruit juice induced a significant decrease of glycemia to normal level in treated diabetic rats. This antihyperglycemic activity of* M. charantia* was demonstrated by several studies [8–11]. It is noteworthy that* M. charantia *fruit juice did not influence glycemia of the control animals, suggesting that the plant juice does not modulate glycemia under normal condition [1]. These observations are in analogy to the results of some investigators who have observed that* M. charantia* failed to influence glycemia in normal control rats [27].

The modulation of the severity or the protection of type 1 diabetes model by leucocytes-derived cytokines has been well reported. The pathogenic role of Th1 cytokines and protective role of Th2 cytokines have been reported in nonobese diabetic (NOD) mice which develop T1D spontaneously and serve as animal models for human T1D [28]. NOD mice represent good model of autoimmune type 1 diabetes [29, 30]. Indeed, Hung et al. [29] have demonstrated the pathological role of Th1 cytokine- (IFN-gamma-) producing cells and IL-12 in autoimmune diabetes in nonobese diabetic mice. On the other hand, other authors [31] have reported that anti-CD20 and IL-10 treatment in NOD mice can modulate the immune functions by upregulating GATA-3 and IL-4 expression and downregulating T-bet and IFN-*γ* expression, which are involved in the pathogenesis of T1D, confirming the protective role of IL-10 in T1D. In fact, IL-10 is known to be a pleiotropic and potent anti-inflammatory and immunosuppressive cytokine that is produced by several types of immune cells including macrophages, dendritic and mast cells, natural killer cells, eosinophils, neutrophils, B cells, CD8+ T cells, CD4+ T cells, and regulatory T cells [20, 24, 32].

In the present study, we observed that STZ-type 1 diabetes in rats induced a significant increase of plasmatic levels of IL-2 and IFN-*γ* (Th1 cytokines) and a decrease of IL-4 (Th2 cytokine) and IL-10 (regulatory cytokine) concentrations. These results are in accordance with those obtained by Saha and Ghosh [33] who have observed a significant increase of inflammatory cytokines* in vivo *after STZ administration in* Wistar* rats, due to increased inflammation in pancreas. In fact, several studies have also reported the regulatory roles of T-helper cell cytokines in multiple low doses of streptozotocin (MLD-STZ) diabetes model, suggesting the pathogenic role of IL-17 and IL-1*β* and protective role of IL-6, IL-10, and IL-4 in MLD-STZ mice [34–37]. For example, Lgssiar et al. [35] have demonstrated the upregulation of Th1 proinflammatory cytokines- (TNF-) alpha and interferon- (IFN-) gamma and downregulation of anti-inflammatory Th2 cytokines interleukin- (IL-) 4 and IL-10 and Th3 cytokine (transforming growth factor, TGF-beta) in islets of multiple low doses of streptozotocin-diabetic male mice. These authors [35] have also demonstrated the protective effect of IL-11, in preventing multiple low doses of streptozotocin diabetes through enhancement of anti-inflammatory responses in islets.

Interestingly, we observed, in the present study, that* M. charantia* fruit juice induced a significant decrease of plasmatic IL-2 and IFN-*γ* concentrations in treated T1D rats as compared to untreated T1D animals. Besides, IL-4 and IL-10 concentrations were significantly increased in treated T1D rats compared with untreated T1D rats. Similar results have been observed by other investigators who have demonstrated anti-inflammatory effect of* M. charantia*, through a decrease in proinflammatory cytokines (IL-1, IL-6, TNF-*α*, and IL-7) and an increase in the secretion of anti-inflammatory cytokines (such as TGF-beta and IL-10) [38, 39]. In order to better appreciate the balance of cytokine production* in vivo*, we calculated the Th1/Th2 ratios. We observed that Th1/Th2 ratios expressed as IL-2/IL4 and IFN-*γ*/IL4 were shifted towards a proinflammatory Th1 phenotype in untreated T1D rats, while these ratios were shifted towards IL-4, a Th2 cytokine, in T1D rats treated with* M. charantia* fruit juice. These observations suggested that* M. charantia* fruit juice promotes a Th2 anti-inflammatory phenotype* in vivo *by decreasing Th1 cytokines and increasing anti-inflammatory Th2 cytokines. Therefore, we can state that the antihyperglycemic effect of* M. charantia* fruit juice may also pass through its decreased action on IL-2 and IFN-*γ* and increased that of Th2 cytokines IL-4 and IL-10. Similar results have been obtained by [40] who have observed that red guava (red-fleshed guava cultivar of* Psidium guajava L*.) exerts antidiabetic effect by suppressing inflammatory (improvement of IL-10 and decrease of TNF-*α*) and oxidative damage caused by diabetes in STZ-induced mice. In the same line, [41] have demonstrated that treating T1D patients with autologous hematopoietic stem cell transplantation (AHSCT) reduced Th1 and Th17 cell expansion and function as well as decreasing IFN-*γ*, IL-2, IL-12p40, and IL-17A levels in the supernatants of peripheral blood mononuclear cell culture.

The concomitant increase of IL-10 with IL-4 levels in T1D rats treated with fruit juice appeared to be normal since IL-10 is known to possess regulatory and anti-inflammatory properties [42, 43]. The present results* in vivo *confirmed our recent findings of the* in vitro* effects of* M. charantia* fruit juice which have induced a Th2 phenotype on human T-lymphocytes [2]. This Th2-immunosuppressive action of* M. charantia *fruit juice could be beneficial to prevent infiltration and destruction of *β*-cells by T-lymphocytes in pancreas of rats and therefore prevent hyperglycemia in T1D rats [13–15]. These observations are in accordance with those of Bao et al. [44] who have shown that* M. charantia* reduces macrophages' and mast cells' infiltration as well as inflammatory cytokine (such as IL-6 and TNF-*α*) expression in epididymal adipose tissues. In contrast, the present results seem to be contradictory with those obtained by Ike et al. [45] who have demonstrated* in vivo* that* M. charantia* pulp has shown an effective immune-stimulatory effect on Th1 cells, producing IFN-*γ*. This discrepancy could be probably due to the mode of administration of the plant juice, as these authors [45] have administrated the juice to the animals by direct intraperitoneal inoculation.

In order to link the chemical contents of the plant juice with its action on T cells differentiation, we carried out the phytochemical screening of fruit juice. We observed that* M. charantia* fruit juice was rich in polyphenols (tannins, flavonoids, and coumarins). Alkaloids and cyanogenic derivatives were undetectable in* M. charantia* fruit juice. These results were similar to our previous recent findings [1]. However, they were in contradiction with those obtained by Johnson et al. [46] who have revealed the presence of alkaloids and free anthracene but not triterpenoids, coumarins, and saponins in* M. charantia* also collected from Benin. This discrepancy could be related to several parameters: parts of plant used, nature of solvent, and mode of preparation, geographical origin, and genetic divergences of the strains used [47]. In this study, we observed that* M. charantia* fruit juice elicited appreciable antioxidant capacity (determined as DPPH free radicals' scavenging) due to the presence of polyphenols (tannins, flavonoids, and coumarins). In fact, it has been shown that flavonoids and tannins act as free radical scavengers [48] and exhibit antioxidant activities, detoxification, and numerous health promoter effects such as anti-inflammatory and antidiabetic [48]. This may confirm the observed anti-inflammatory and antioxidant activity of* M. charantia* fruit juice in this study.

## 5. Conclusion

The novelty of the present study resides in the fact that the present results* in vivo* confirm our recent findings* in vitro* on the Th2-anti-inflammatory and immunosuppressive activity of saponins-depleted* M charantia* fruit juice. Moreover, our results confirm the antihyperglycemic and antioxidant activity of* Momordica charantia*. Thus, the Th2-anti-inflammatory property of* Momordica charantia *fruit juice may contribute to inhibiting deleterious effect of autoreactive T-lymphocytes on *β*-cells in type 1 diabetes, and this needs to be furthermore investigated in animals and human beings. Future studies are required to evaluate whether the protective action of* Momordica charantia* fruit juice reverts after interruption of the treatment or if it is maintained. In a similar manner commencing a treatment with* Momordica charantia* under therapeutic regime to rats with established diabetes could be interesting.

## Figures and Tables

**Table 1:** Ratios of Th1 and Th2 cytokine concentrations in plasma.

	IL-2/IL-4	IFN-*γ*/IL-4
Control rats	0,37	9,36
T1D rats	1,51^*∗*^	35,15^*∗*^
Control rats + FJ	0.36	9.34
T1D rats + FJ	0,33^*δ*^	11,99^*δ*^

Values are ratios of mean concentrations of plasma Th1/Th2 cytokines. *n* = 10 rats in each animal groups. ^*∗*^*p* < 0.05 indicates significant difference between type 1 diabetic (T1D) rats and control rats and ^*δ*^*p* < 0.05 indicates significant difference between untreated T1D rats and treated T1D rats with *M. charantia* fruit juice (FJ).

**Table 2:** Phytochemical compositions of *Momordica charantia* fruit juice.

Chemical compounds class	Tests	*Momordica charantia* fruit juice
Alkaloids	General test: Dragendorff reagent	−
	Extraction: Mayer reagent	−
Tannins	Few drips of FeCl3, 1%	++
Flavonoids	Adding four drips of HCl 5% to 1 ml of juice	++
Saponins	Foam index (FI) of diluted aqueous decoction (positive if FI ≥ 100, meaning foam height ≥ 1 cm)	− (FI < 1 cm)
Triterpenoids	Liebermann-Burchard reaction (acetic anhydride-sulfuric acid 50 : 1)	+
Mucilages	Viscosity study (in absolute ethanol)	+
Coumarins	Addition of 0.5 ml of NH4OH 10%	++
Anthraquinones	Addition of 1 ml NH4OH 25% + 1 ml NaOH	−
Steroids	Acetic anhydride-chloroform + concentrated sulfuric acid	+
Cyanogenic derivates	Grignard reaction soaked paper with picric acid 5%	−

Chemical compounds of *Momordica charantia* fruit juice. The phytochemical analysis was performed as described in *Materials and Methods*. (++) high, (+) low indicate the presence of the compounds in the plants; (−) indicates the absence of compound in juice.
